# Time series insights from the shopfloor: A real-world dataset of pneumatic pressure and electrical current in discrete manufacturing

**DOI:** 10.1016/j.dib.2024.110619

**Published:** 2024-06-10

**Authors:** Žiga Stržinar, Boštjan Pregelj, Janko Petrovčič, Igor Škrjanc, Gregor Dolanc

**Affiliations:** a “Jožef Stefan” Institute, Jamova cesta 39, SI-1000 Ljubljana, Slovenia; b University of Ljubljana Faculty of Electrical Engineering, Tržaška cesta 25, SI-1000 Ljubljana, Slovenia

**Keywords:** Time series analysis, Clustering, Segmentation, Classification, Industry, Labeled, Multivariate

## Abstract

Gathered from a real-world discrete manufacturing floor, this dataset features measurements of pneumatic pressure and electrical current during production. Spanning 7 days and encompassing approximately 150 processed units, the data is organized into time series sampled at 100 Hz. The observed machine performs 24 steps to process each unit. Each measurement in the time series, is annotated, linking it to one of the 24 processing steps performed by the machine for processing of a single piece. Segmenting the time series into contiguous regions of constant processing step labels results in 3674 labeled segments, each encompassing one part of the production process. The dataset enriched with labels facilitates the use of supervised learning techniques, like time series classification, and supports the testing of unsupervised methods, such as clustering of time series data.

The focus of this dataset is on an end-of-line testing machine for small consumer-grade electric drive assemblies (device under test – DUT). The machine performs multiple actions in the process of evaluating each DUT, with the dataset capturing the pneumatic pressures and electrical currents involved. These measurements are segmented in alignment with the testing machine's internal state transitions, each corresponding to a distinct action undertaken in manipulating the device under observation.

The included segments offer distinct signatures of pressure and current for each action, making the dataset valuable for developing algorithms for the non-invasive monitoring of industrial (specifically discrete) processes.

Specifications Table

**Table:** 

Subject	Applied Machine Learning
Specific subject area	Time series analysis of shopfloor industrial data for use in classification, clustering and segmentation
Type of data	Raw
Data collection	A pneumatic pressure sensor was installed at the pneumatic pressure line powering the machine. An electrical current sensor was installed to measure the aggregated consumption of machine actuators. Both sensors were sampled at 100Hz for the duration of data acquisition. The machines internal state machine, which tracks the various steps involved in processing a single piece, was monitored for events indicating transitions between processing steps. The timestamps were recorded and synchronized with the pneumatic pressure and electrical current measurements.
Data source location	An end-of-line testing machine for consumer-grade small electric drive assembly at large European supplier of small electric drives for consumer appliances, the automotive sector, and e-bikes. Data collection performed by “Jožef Stefan” Institute, Jamova cesta 39, SI-1000 Ljubljana, Slovenia.
Data accessibility	Repository name: Mendeley Data Data identification number: 10.17632/ypzswhhzh9.2 Direct URL to data: https://data.mendeley.com/datasets/ypzswhhzh9 Instructions for accessing these data: Data is freely available for download. Data can be accessed by reading included .csv files or through using Python's pickle module.
Related research article	-

## 1 Value of the Data

• Industrial datasets obtained from real world production lines, such as the one presented here, are of great value. They enable researchers to develop and validate their algorithms under realistic conditions, which is often not possible with synthetic, simulated, laboratory, or non-industrial data.
• The primary value of this dataset [1] is to enable the wider time series research community to develop, test and evaluate their algorithms on real world industrial time series data. This dataset is particularly useful for algorithms dealing with time series segmentation, classification, and clustering.
• A key feature of this dataset is the ground truth annotation of current processing steps for each timestamp. These annotations can be used to divide the time series into contiguous chunks, each corresponding to a single processing step. These segments can then be used for clustering and classification, allowing for empirical evaluation of these algorithms.
• Open datasets such as the one presented here, enable the time series research community to compare existing and new algorithms. Using open datasets enhances reproducibility of research results, leading to higher quality research.
• Manufacturers are typically very cautious of publishing datasets from their production lines due to confidentiality concerns. However, we have reached an agreement with one manufacturer to publish this dataset, by choosing measured variables which do not reveal any confidential details of the manufacturing process. Despite this, the dataset remains valuable for the wider time series analysis community.
• We consider Time Series Segmentation to be a crucial task in Time Series Analysis. While much segmentation research focuses on finding points where the system changes patterns (switch points [[2], [3], [4]]) in discrete manufacturing processes, it is more interesting to identify individual tasks. This dataset, alongside similar datasets from other production processes, can be used to train segmentation algorithms. Possible applications of machine learning algorithms developed using this dataset include: 1) automatic segmentation of time series in discrete manufacturing processes [[5], [6]], clustering of segments [[7], [8], [9], [10]], and detection of anomalous processing patterns, and 2) evaluation of time series classification (TSC) algorithms, making them more useful in industrial settings for predictive maintenance [11], health monitoring, quality control [12], energy load analysis [13], pollution analysis, etc.

## 2 Background

Our research [14] focuses on the application of machine learning methods to problems faced by manufacturers, the energy sector etc. One open research topic is monitoring of industrial processes using noninvasive measurements i.e. without adding additional sensors within the machine, which might void the warranty.

Several open datasets for evaluating time series machine learning algorithms exist. The most impactful work in this field has been the UCR Archive [15], a collection of over a hundred time series datasets primarily focused on time series classification [16,17]. While the Archive includes datasets from a variety of domains, industrial datasets captured at real-world production lines are lacking. Our work aims to fill this gap.

On the factory shop floor, monitoring industrial processes is crucial for achieving increased productivity, detecting and eliminating bottlenecks, and reducing downtime. An effective monitoring system should be capable of detecting and recognizing events at observed machines. Our proposed analysis pipeline involves several steps: first, signals related to the machine's operation are gathered. These time series signals are then segmented using machine learning algorithms, with each segment ideally representing a single task performed by the machine. The segments can then be classified into task identifiers (e.g., using a time series classification algorithm). This stream of task identifiers provides process insights. For data acquisition, two approaches are considered: focusing on key process variables or using more general signals common in industrial plants. The general approach results in more versatile algorithms that can be transferred across various industrial processes.

The dataset described in this paper aligns with the guidelines outlined above. We chose to measure pressure and electrical signals, which are ubiquitous on the shop floor and not specific to any particular machine.

A similar but smaller dataset, obtained in a laboratory setting, was used in [14] for developing a time series classification algorithm. This dataset was combined with the UCR Archive [15] for the development and evaluation of the classification algorithm. We believe that the dataset presented here can be used in the future to further develop and improve time series segmentation, classification, and clustering algorithms.

## 3 Data Description

**Fig. 1:**
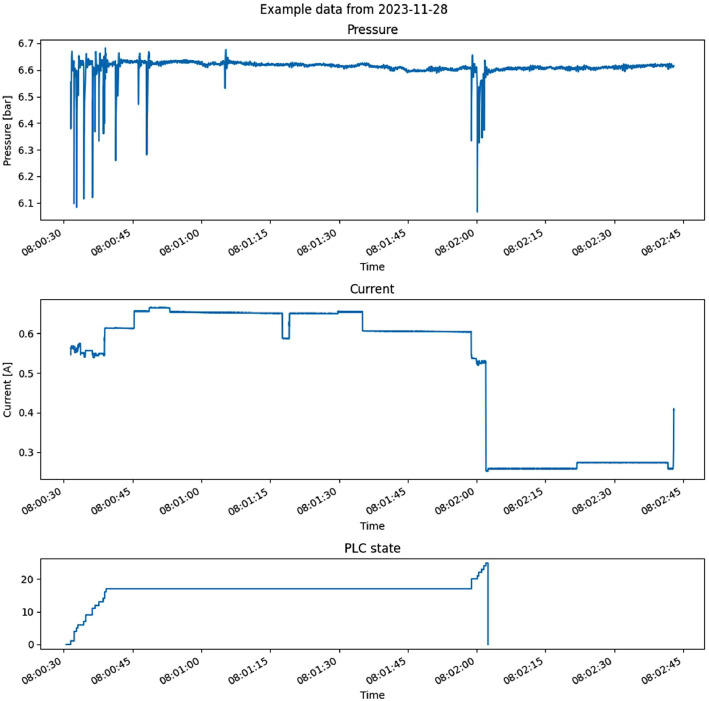
Examples of pressure, current and PLC state file contents.

The data collected is part of a monitoring system supervising the end-of-line station. The purpose of the monitoring system is to detect abnormalities in the testing station's operation, such as missed operating steps, drifts in the speed of operation, and other issues. An example of the data gathered is shown in Fig. 1.

This monitoring system has access to the internal PLC states, which it uses to segment the pressure and current signals into segments representing each PLC state—i.e., the tasks performed by the testing station when testing a single DUT (Device Under Test). In general, a monitoring system might not have access to the PLC's internal states and would therefore need to apply an algorithm to segment the signals.

After performing segmentation, the monitoring system can conduct additional time series analysis operations, such as finding groups of similar segments using clustering algorithms.

The dataset presented in this paper aims to demonstrate such a processing pipeline and enable the development of time series analysis methods for online monitoring of industrial processes. Since the originating test station is part of a discrete production line, this dataset is of particular interest to researchers focusing on discrete manufacturing (as opposed to batch or process manufacturing).

### 3.1 Data files

The published dataset contains:

1. 468 CSV files – raw data
2. Two .pkl files – for easier loading and processing in Python
3. Two Jupyter Notebooks demonstrating how to load and explore both CSV and .pkl data

This dataset provides insights into the behavior and operational transitions of the testing machine, meticulously recording how the machine handles various testing phases, rather than detailing the specifics of the electric drive assemblies (Device Under Test – DUT).

### 3.2 Raw data

The raw data is available in 468 CSV files in folder Data/Raw. The name of each file is comprised of:

1. Prefix
2. Timestamp
3. Suffix

The prefix of each file reveals the nature of the file:

**Table:** 

Prefix	Meaning
Dump_long_100Hz_hall_sensor_	Current measurements using Hall sensor
Dump_long_100Hz_line_pressure_	Line pressure measurements
Dump_long_PLCstate_dt	Log of PLC state changes

The timestamp included in each file name follows the convention:

­ Four-digit year
­ Two-digit month (leading 0)
­ Two-digit day of month (leading 0)
­ Underscore (_)
­ Two-digit hour of day (leading 0)
­ Two-digit minute (leading 0)
­ Two-digit second (leading 0)

Example: 20231127_100923 denotes 27^th^ November 2023 at 10:09:23.

The suffix is always .csv.

The raw data in .csv files contains pressure and current measurements sampled at 100Hz. Each file contains data in two columns, delimited by a semicolon. The first column indicates the timestamp without the date, the second column contains the measured value – pressure or current. The first line of each file is the header.

The .csv files containing the log of PLC state changes are not sampled. Just the timestamps of state changes are recorded. Therefore, each PLC state csv file is typically much smaller than the pressure and current files.

No guarantees are given as to the splitting of data into subsequent csv files, however, typically, a new file is started during a longer pause in the manufacturing process.

### 3.3 Python-ready files

Today, Python is the programming language of choice for many in the machine learning community. In order for our dataset to be accessible to as large an audience as possible, we have prepared the data in a Python-friendly format.

The pickle library included in Python enables serialization and deserialization of Python objects. This enables complex objects to be serialized, saved to disk, loaded at a later time and deserialized without loss of data. The practical implications for us are that we can load the raw data described above, extract segments based on the PLC state records, and save the result for further use by a wide audience.

We provide two .pkl files in Data/Pickled folder: segmented_current.pkl and segmented_pressure.pkl.

Each file contains pre-segmented and labeled time series. Each segment (and associated label) corresponds to raw data between two changes in PLC state.

To obtain .pkl files, we have loaded all the CSV files and segmented the time series wherever the PLC state changes.

By deserializing each .pkl file, the user gets a Python dictionary containing two keys: data and labels. Data contains a list of 3674 pandas Series objects. Labels contains a list of 3674 integers – these are the PLC state labels of corresponding data segments. Each pandas Series object is indexed by the timestamps.

### 3.4 Jupyter Notebooks for demonstration

Jupyter Notebooks are a common development and presentation tool used in python development, especially in machine learning. They contain code, in-line results, which can even be dynamic (can respond to user-inputs), and descriptive text. Jupyter Notebooks are a great way to demonstrate python libraries, analysis results etc.

We provide two Jupyter Notebooks to demonstrate: 1) loading the dataset, both in CSV and .pkl formats, and 2) exploring the content of the dataset.

### 3.5 Dataset

In Fig. 1, we plot the content of three CSV files (corresponding to pressure, current, and PLC state) with a matching timestamp in their names.

The bottom plot in Fig. 1 shows the changing of the PLC state from 0 to 25, and back to 0. Careful investigation will reveal that some states in the range from 0 to 25 are missing. This is due to the internal logic of the observed PLC which skips some states.

The top plot shows the pressure measurements in Bars. We see four distinct regions of the pressure signal. In the first part, the measurements change rapidly; in the second part, the pressure remains relatively constant; in the third part, the measurements are again more spread out; and in the last part, again the pressure measured is more constant. Comparing this with the bottommost plot, we see that regions of variable pressure align with changes in PLC state.

The middle plot shows current measurements. Again regions where PLC state changes, the current changes as well. However, unlike pressure, current also has significant changes in amplitude during the long period when pressure and PLC state are constant (see 08:01:20). During this period, the observed machine is performing actual measurements of the DUT, which involves engaging relays, powering the DUT, etc. The inner workings of the test procedure are not observable in the PLC state variable and in pressure measurements.

**Fig. 2:**
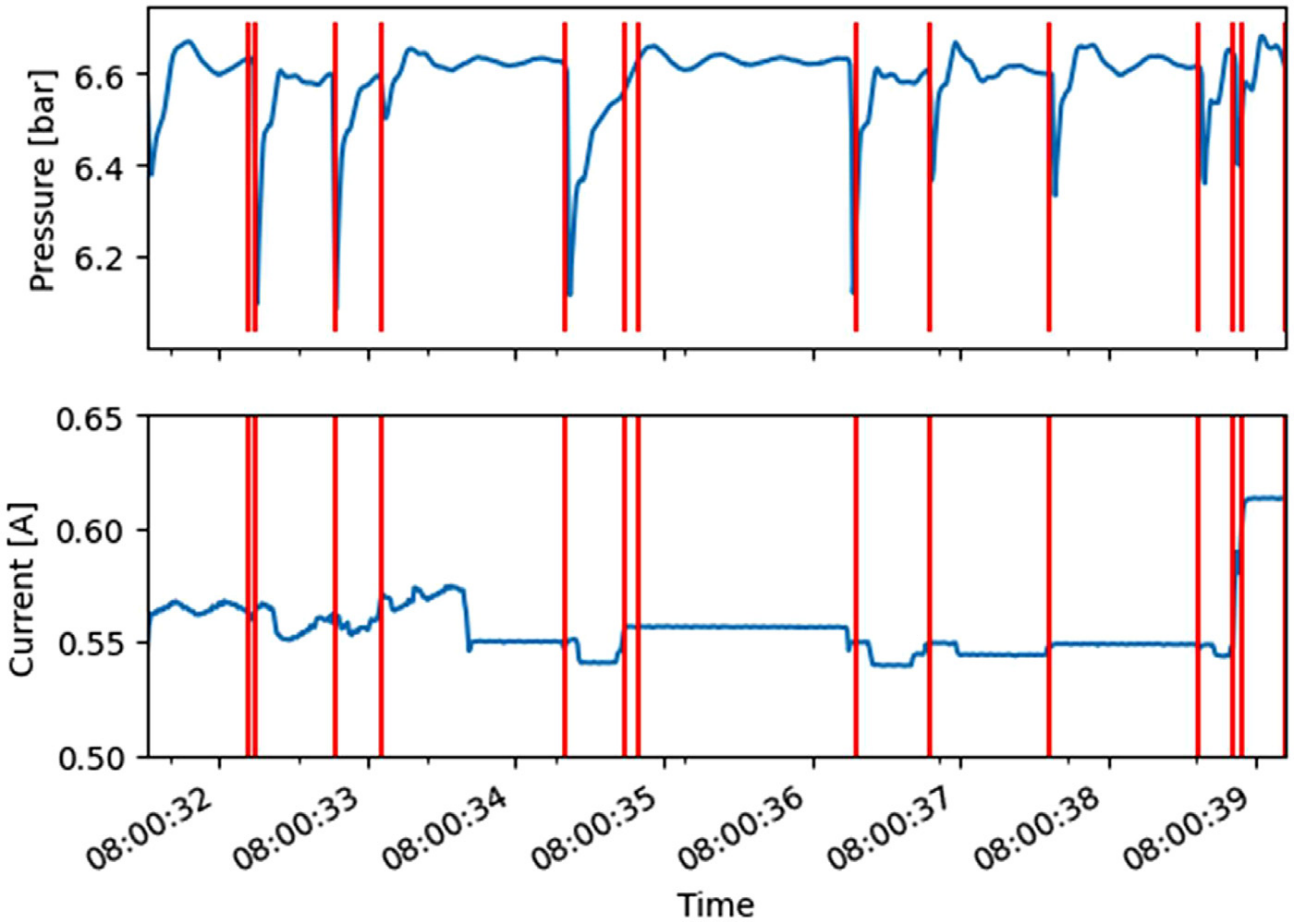
Examples of pressure and current signals. Vertical red lines indicate points where PLC state changes value.

In Fig. 1, we have shown the contents of an entire file. Due to the nature of the observed machine, most of PLC state changes occur in a small portion of the entire time series shown. In Fig. 2, we zoom in to this ‘dynamic’ region to better demonstrate the effect of PLC state changes on the observed signals. We show pressure and current measurements in top and bottom plots. Both plots include red vertical lines corresponding to timestamps when the PLC state changes value.

We can observe that changes in PLC state have a direct effect on pressure measurements, namely, the pressure drops and then recovers roughly to the previous level. This is a result of pneumatic actuators firing when PLC issues the command. The line pressure temporarily drops, and the pressure control system then increases the pressure back to the desired levels. Changes in PLC state are also reflected on the current measurements. Fig. 2 indicates that actuators have characteristic pressure and current signatures, which can be analyzed and exploited in future work, for example to obtain class prototypes [8,10,18].

**Fig. 3:**
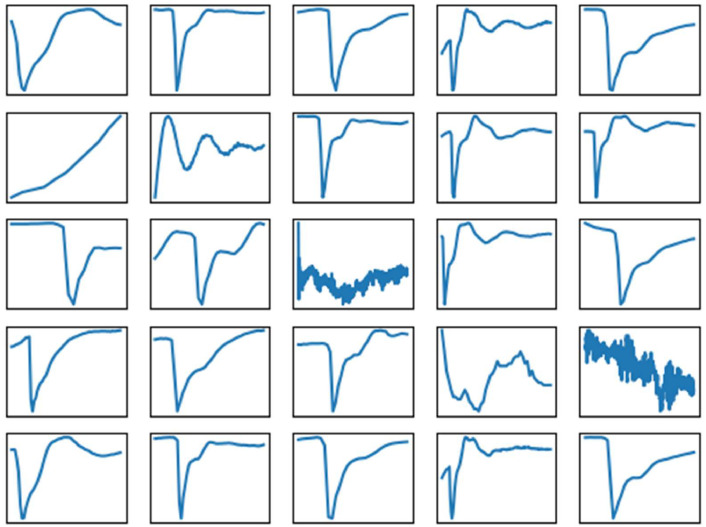
One example of pressure signal from each of PLC states. Axis labels and ticks are omitted in favor of larger plots.

**Fig. 4:**
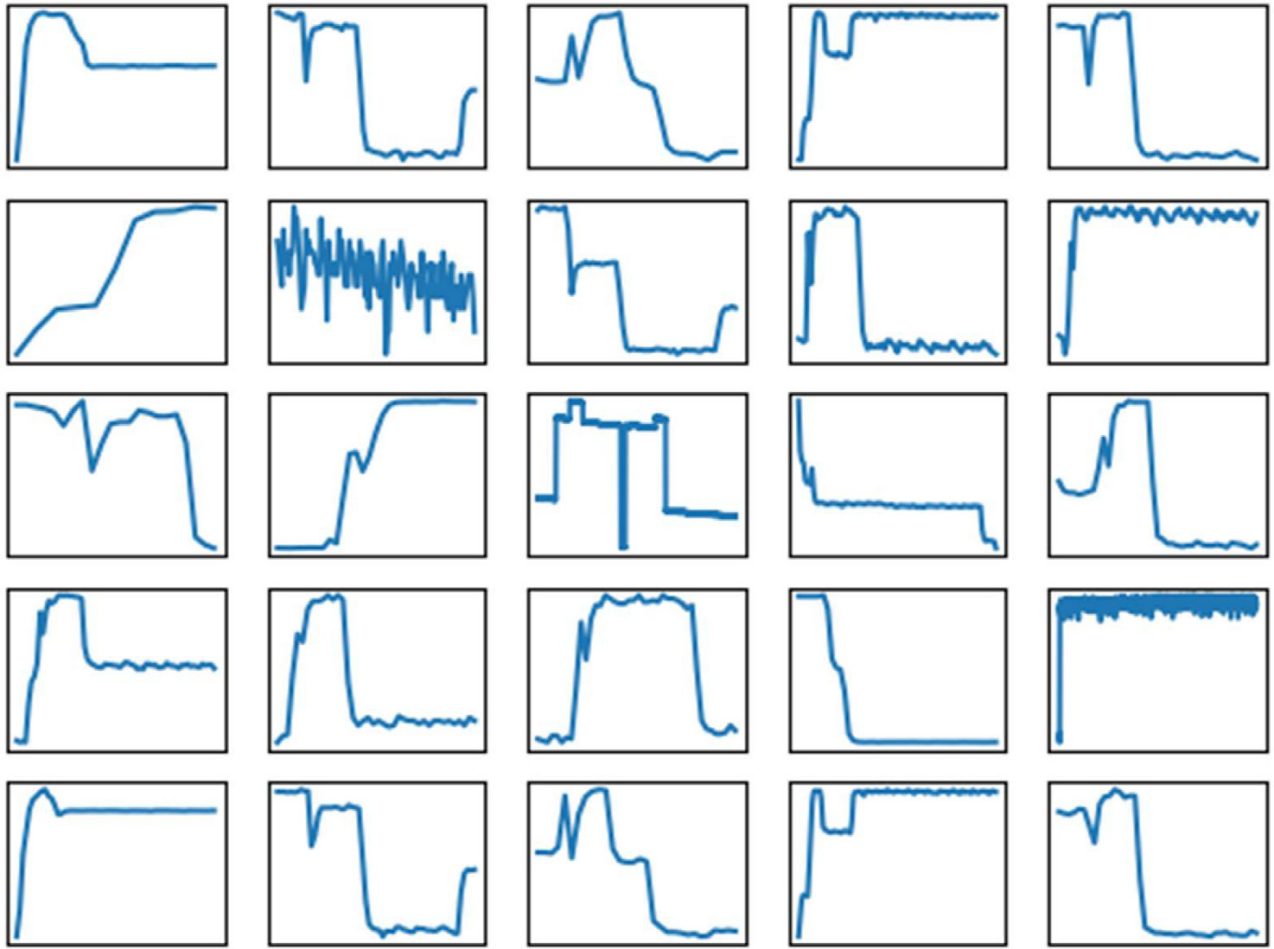
One example of current signal from each of PLC states. Axis labels and ticks are omitted in favor of larger plots.

**Fig. 5:**
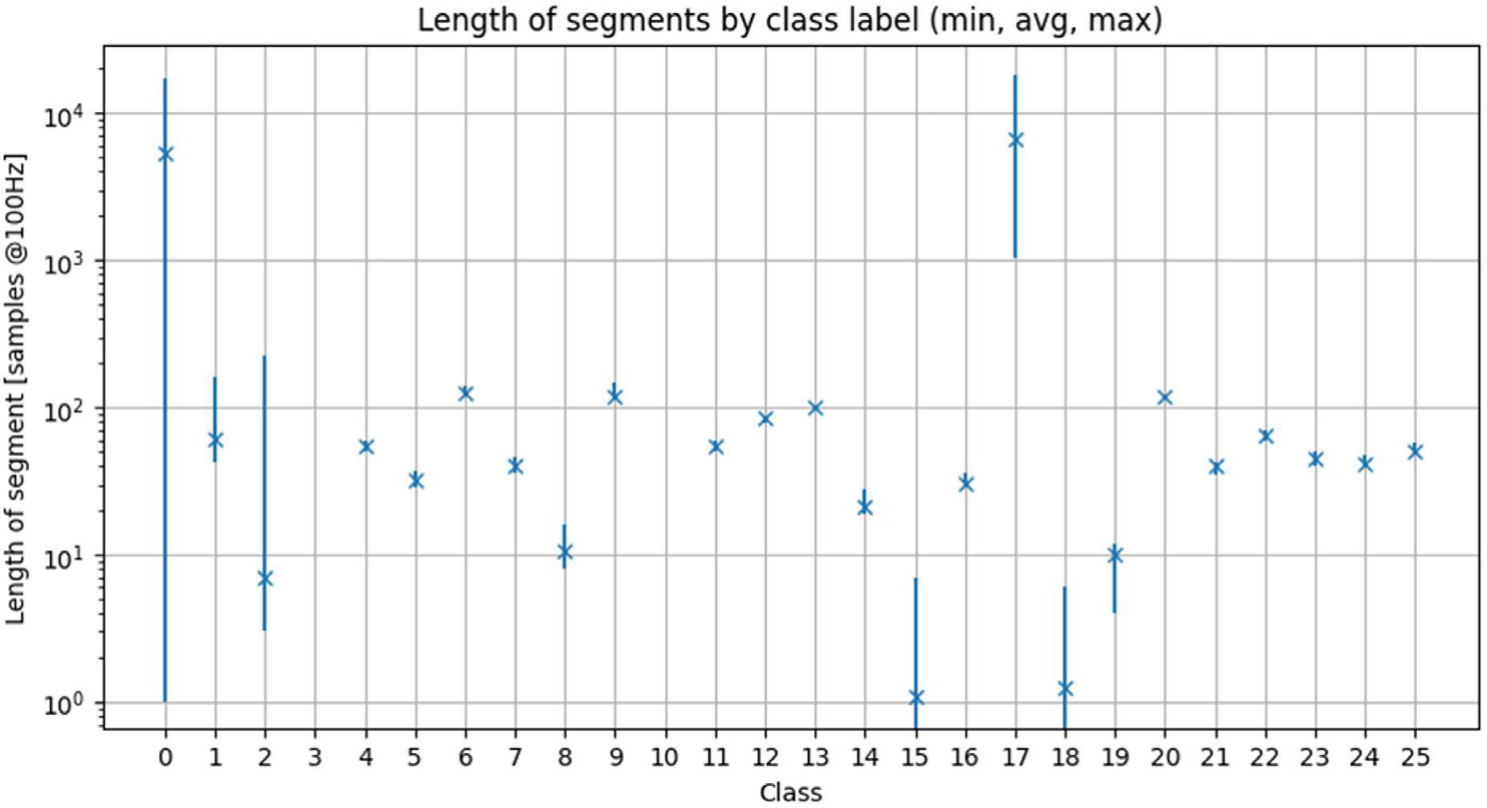
Lengths of samples associated with each class label. X symbols indicate the mean, vertical lines show the full range.

In Figs. 1 and 2, we have shown raw data. Data can be segmented at PLC state changes (i.e., red lines in Fig. 2). We have done this and saved the results in pkl files. Figs. 3 and 4 demonstrate examples of measurements associated with each PLC state label. In both figures, the X and Y axis ticks and labels are omitted in favor of larger plots. Although each segment in Figs. 3 and 4 seems of equal length, Fig. 2 has shown that their length varies significantly. The variability of segment lengths is also demonstrated in Fig. 5.

## 4 Experimental Design, Materials and Methods

### 4.1 System description

Dataset has been gathered at an end-of-line testing station. The station is designed to test electric drives. The station processes one piece at a time. The duration of the test procedure depends on the DUT type being tested and the exact procedure set up at the control system, but in general it takes between 1 and 3 minutes to evaluate a single DUT.

**Fig. 6:**
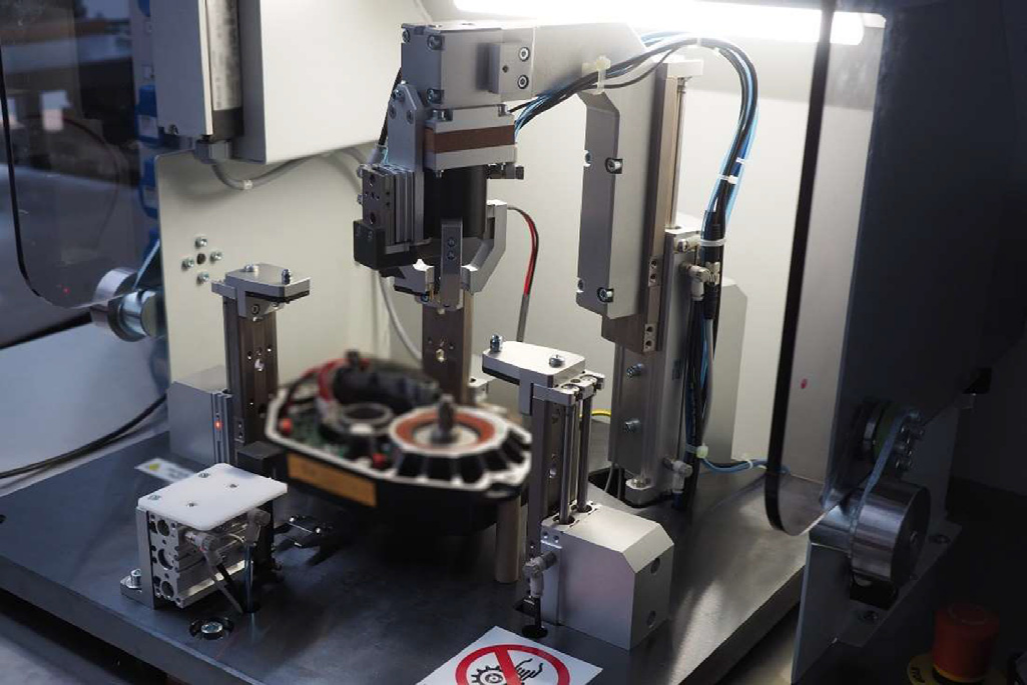
Test station. The piece being tested is intentionally blurred.

A photograph of the test station is given in Fig. 6. The photograph depicts the electric drive in central position, it is surrounded by two horizontal gripping mechanisms and a vertical arm with a gripper.

The focus of the test station is to validate the communication and performance characteristics of the DUT. The scope of our dataset is wider – we observe the test station itself, not the electric DUT. In line with our other research work, we are interested in the operation and performance of the test station. In the data, we can observe several actions before and after the main test procedure. Those actions are crucial to the performance of the test station - see Figs. 1 and 2.

### 4.2 Data acquisition

**Fig. 7:**
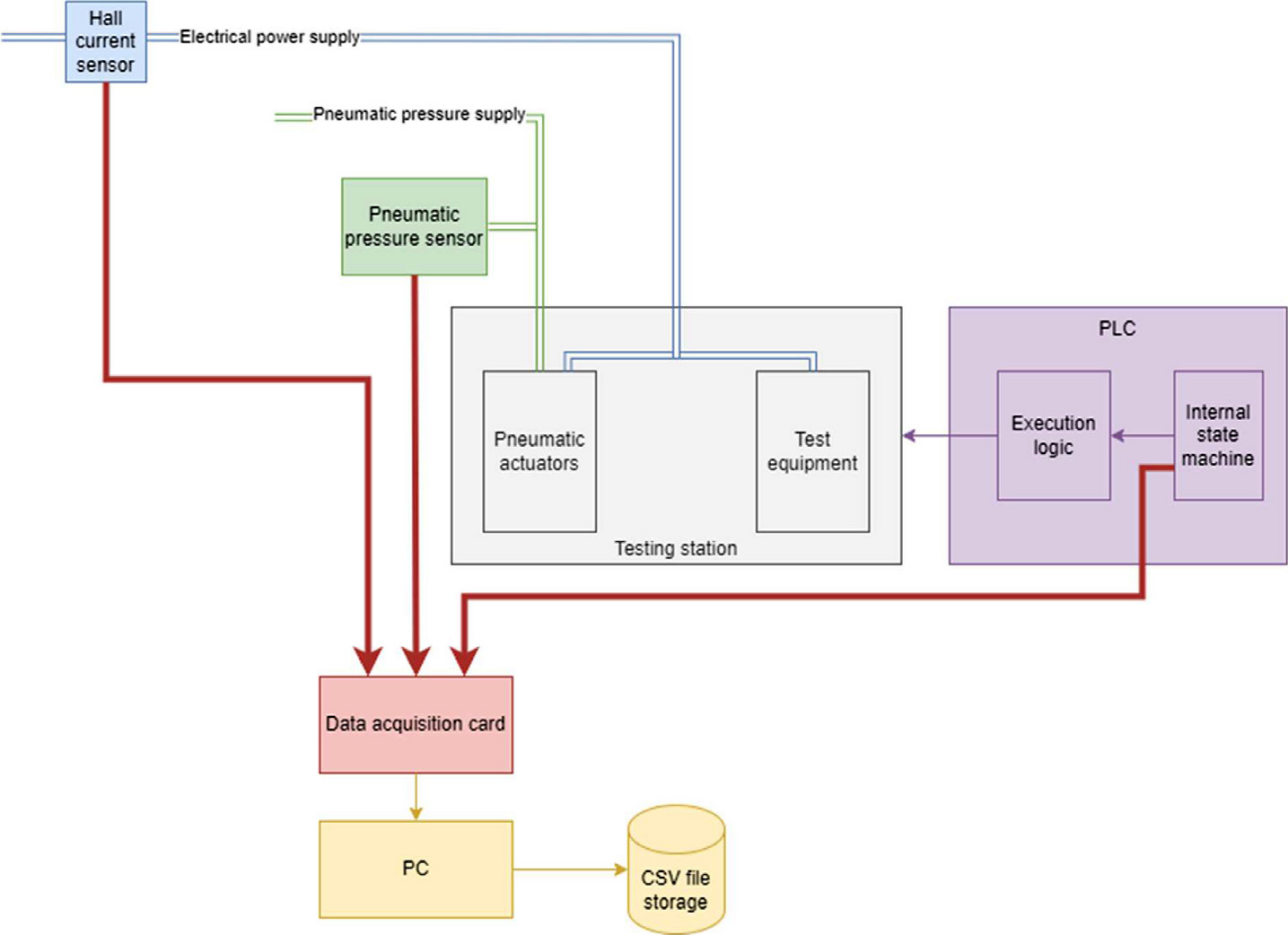
Data acquisition system.

Fig. 7 depicts the Data Acquisition System, installed on the end-of-line product testing station. The testing station shown in gray is comprised of test equipment and pneumatic actuators. The test equipment is tasked with performing the tests of the DUT, wheras the pneumatic actuators are tasked with positioning the DUT and test equipment in place. The testing station is controlled by a Programmable Logic Computer (PLC), which has an internal state machine governing the test procedure. The state machine ensures the correct sequence of positioning events of the DUT prior and after the test procedure. The internal state machine, through the use of execution logic, controls the testing station.

The testing station is powered by electric current and pneumatic pressure. Both quantities are measured using a Hall current sensor and a pneumatic pressure sensor, respectively. The signals are conditioned using analog filtering setup and acquired via NI-USB 6215 data acquisition module. In-house developed software is used for automatic raw data logging and file notation.

The internal state machine, the pneumatic pressure sensor and the hall current sensor are connected to a data acquisition card, which inturn in connected to a PC running data acquisition software. The software stores measurements into CSV files.

Pneumatic pressure and electrical current are sampled at 100Hz. The PLC state is sampled only at value changes (state transitions).

## Limitations

Not applicable.

## Ethics Statement

This work does not involve human subjects, animal experiments, or any data collected from social media platforms.

## CRediT authorship contribution statement

**Žiga Stržinar:** Conceptualization, Methodology, Software, Validation, Formal analysis, Investigation, Data curation, Writing – original draft, Writing – review & editing, Visualization. **Boštjan Pregelj:** Conceptualization, Methodology, Software, Validation, Formal analysis, Investigation, Resources, Data curation, Writing – review & editing, Supervision, Project administration, Funding acquisition. **Janko Petrovčič:** Conceptualization, Writing – review & editing, Funding acquisition. **Igor Škrjanc:** Conceptualization, Methodology, Resources, Writing – review & editing, Supervision. **Gregor Dolanc:** Conceptualization, Methodology, Resources, Writing – review & editing, Funding acquisition.

## Data Availability

Pneumatic Pressure and Electrical Current Time Series in Manufacturing (Original data) (Mendeley Data).
